# Salvira™ Does Not Modulate Estrogen Receptor: Reassessing *Salvia officinalis* Proposed Phytoestrogenic Properties

**DOI:** 10.3390/nu18213497

**Published:** 2026-10-24

**Authors:** Rossana Iannitti, Giada Ceccarelli, Valentina Melfi, Mariano Licciardi, Francesco Montalbano, Desirée Bartolini, Elisa Menicali, Alice Coletti, Chiara Pennetta

**Affiliations:** 1 S&R Farmaceutici SpA, 06083 Bastia Umbra, Italy; 2 Department of Biological, Chemical and Pharmaceutical Sciences and Technologies (STEBICEF), University of Palermo, Via 8 Archirafi 30, 90123 Palermo, Italy; 3 Technology Scientific S.r.l., Via del Quarnaro 14, 90144 Palermo, Italy; 4 Dephi STARLab, Department of Pharmaceutical Sciences, University of Perugia, 06123 Perugia, Italy; desiree.bartolini@unipg.it (D.B.);

**Keywords:** *Salvia officinalis*, estrogenic effect, menopause, vasomotor symptoms, MCF-7, Erα

## Abstract

**Background/Objectives**: *Salvia officinalis* L. is widely used in food supplements, particularly those intended to support women’s health and alleviate menopausal vasomotor symptoms. However, the extent to which estrogen receptor signaling contributes to its reported clinical benefits remains unclear. Therefore, evaluating its potential endocrine activity is a key aspect of its toxicological characterization and overall safety assessment. The present study aimed to investigate the potential estrogenic effects of a standardized Italian *Salvia officinalis* L. extract (Salvira™), as well as its biological effects on estrogen-responsive breast cancer cells (MCF-7). **Methods**: In vitro enzymatic assays and estrogen receptor alpha (ERα) agonist- and antagonist-mode experiments were performed to assess its ability to modulate ERα signaling. Moreover, cell viability, proliferation, and clonogenic capacity were assessed in MCF-7 cells following exposure to increasing concentrations of the extract. **Results**: The extract did not exhibit estrogenic activity in all experimental dose–response assays. In MCF-7 cells, treatment with the sage extract did not produce an overall effect on cell viability or proliferative capacity. Moreover, no impairment of long-term clonogenic potential was observed at the tested concentrations. **Conclusions**: Under the experimental conditions investigated, the present findings suggest that the standardized *Salvia officinalis* L. extract showed no evidence of estrogenic activity and did not induce relevant ERα-mediated biological effects in estrogen-responsive MCF-7 cells. These findings contribute to the characterization of the endocrine safety profile of the extract, supporting its safe use as an ingredient in food supplements.

## 1. Introduction

Menopause affects approximately 1.2 billion people worldwide, with a mean age of onset of 51 years in Western populations, though significant variability exists across ethnic groups and geographic regions [1]. The menopausal transition is associated with a plethora of symptoms which impose a substantial burden on quality of life and functional capacity. Vasomotor symptoms (VMSs) are the cardinal symptoms of menopause, alongside other key health concerns such as raised risk of heart disease and osteoporosis, sleep disturbances, vaginal dryness and mood changes [2,3].

VMSs, primarily manifesting as hot flashes and night sweats, represent the most prevalent and clinically significant complaints associated with the menopausal transition, affecting approximately 70–80% of women in Western populations [4]. Despite the high prevalence and substantial burden of these symptoms, evidence consistently indicates that VMSs remain significantly undertreated in clinical practice [5].

Recently, nutritional supplementation has emerged as a natural alternative solution for menopause management, particularly among women who are unwilling or unable to use conventional hormonal therapies due to contraindications, personal preference, or residual concerns regarding hormone therapy safety debates [6,7,8]. The supplements currently available in this setting are broad and heterogeneous. Phytoestrogens, plant-derived compounds with structural and functional similarities to endogenous estrogens, represent the most extensively studied category. Isoflavones derived from soy and red clover have demonstrated modest but statistically significant reductions in vasomotor symptom frequency and severity in several randomized controlled trials and meta-analyses, with effects attributable to selective agonist activity at estrogen receptor beta (ERβ) [9,10,11]. While phytoestrogen-based supplements are frequently perceived as inherently safe by virtue of their natural origin, their potential estrogenic activity may limit their use, particularly in individuals at risk of hormone-sensitive cancer [12,13]. In light of these limitations, there is still a need to investigate novel categories of dietary supplements acting through alternative mechanisms with a high safety profile.

*Salvia officinalis* L. (common sage), a member of the Lamiaceae family with a longstanding history of ethnopharmacological use in Mediterranean traditional medicine, has attracted growing scientific interest as a candidate botanical intervention for the management of VMSs [14,15,16]. The empirical basis for its use derives from traditional medicinal practices, recent mechanistic findings, molecular characterization and clinical evidence.

Beyond its medicinal applications, *Salvia officinalis* has a long history of use as a culinary herb and traditional food ingredient, particularly in Mediterranean countries. Its extensive consumption as part of the human diet supports its suitability for food applications and provides a valuable bridge between traditional dietary use and contemporary functional nutrition. In recent years, sage extracts have been increasingly incorporated into functional foods and nutraceutical formulations as natural sources of antioxidant compounds and as alternatives to synthetic preservatives. This evolution from traditional culinary use to evidence-based nutritional applications has been driven by growing scientific recognition of the biological properties of sage phytochemicals [17,18]. Among these constituents, diterpenoids such as carnosic acid and carnosol, together with rosmarinic acid and selected flavonoids, are believed to contribute to the pharmacological effects of sage relevant to menopausal symptomatology. Rosmarinic acid is generally recognized as one of the major water-soluble bioactive constituents of sage, whereas carnosic acid and carnosol contribute substantially to its antioxidant capacity [19]. Furthermore, the principal phenolic compounds of sage are absorbed in the gastrointestinal tract and undergo extensive biotransformation into conjugated metabolites, which are also believed to contribute to the biological effects observed in vivo. Consequently, the nutritional and physiological relevance of sage-derived ingredients depends not only on their phytochemical profile but also on the bioavailability and metabolic fate of these compounds following ingestion [19]. Taken together, the long-standing dietary consumption of sage, combined with increasing evidence supporting the bioaccessibility and physiological activity of its major phenolic constituents, provides a strong rationale for investigating standardized sage extracts as safe and biologically relevant ingredients for functional foods and nutraceutical interventions targeting women’s health, including the management of menopausal vasomotor symptoms. 

Unlike classical phytoestrogens, the mechanism of action of sage remains incompletely understood and may involve both estrogen-dependent and estrogen-independent pathways.

The pharmacological activity of *Salvia officinalis* L. relevant to menopausal symptomatology is attributable to a complex mixture of bioactive components. Diterpenoids, particularly carnosic acid and carnosol, the phenolic glycoside rosmarinic acid, and the flavonoids luteolin, apigenin, and hispidulin collectively constitute the principal phytochemical complex of sage leaf extracts. Rosmarinic acid has been investigated for its capacity to inhibit acetylcholinesterase and to modulate GABAergic and serotonergic neurotransmission, pathways of relevance given the established role of central serotonergic dysregulation in the thermoregulatory instability underlying hot flushes [20]. Additionally, sage may help reduce both hot flushes and night sweats because of its antiperspirant effects [21]. Despite the well-documented clinical efficacy of *Salvia officinalis* L. in reducing the frequency and intensity of vasomotor symptoms in menopausal women, unlike classical phytoestrogens, the mechanism of action of sage remains incompletely understood and may involve both estrogen-dependent and estrogen-independent pathways [14,22].

Several studies have reported estrogenic activity in aqueous–ethanolic extracts of sage, attributing this effect primarily to flavonoid constituents such as luteolin-7-O-glucuronide and luteolin-7-O-glucoside, which appear to interact with estrogen receptors in a manner analogous to classical phytoestrogens [23,24]. Conversely, other investigations have failed to identify a consistent and reproducible estrogenic signal, and the contribution of isoflavones to sage’s overall bioactivity has been shown to be negligible [23,25]. Furthermore, the marked variability in phytochemical composition across different sage preparations, largely dependent on extraction methods and plant material sourcing, significantly complicates the interpretation and cross-study comparison of pharmacodynamic data [23,26]. Alternative or complementary mechanisms, including the modulation of thermoregulatory pathways independent of estrogen receptor binding, cannot be excluded and remain insufficiently explored. Taken together, these uncertainties underscore the urgent need for mechanistic studies combining standardized botanical extracts with receptor-binding assays to definitively characterize the pharmacological profile of *Salvia officinalis* L. and establish whether its therapeutic effects are mediated through estrogenic, non-estrogenic, or mixed pathways.

Given the ongoing uncertainty regarding the contribution of estrogen receptor signaling to the clinical effects of sage, the present study aimed to investigate the potential estrogenic agonist activity of a standardized Italian *Salvia officinalis* extract, thereby contributing to a clearer characterization of its estrogen-dependent and estrogen-independent biological mechanisms.

## 2. Materials and Methods

### 2.1. Reagents

The standardized *Salvia officinalis* L. extract used in this study was supplied by Nutraceutica S.r.l. (Bologna, Italy) as a standardized dry extract obtained from sage leaves. According to the technical specifications provided by the manufacturer, the extract is produced using water as the extraction solvent, yielding a dry extract with a drug–extract ratio (DER) of approximately 4:1, standardized to contain ≥1.5% total hydroxycinnamic derivatives (expressed as rosmarinic acid) and 0.5–1% rosmarinic acid. Maltodextrin is used as an excipient in the final product.

The use of water extraction was selected because it is a food-grade, environmentally friendly solvent suitable for the production of ingredients intended for food supplements. Furthermore, aqueous extraction preferentially concentrates hydrophilic phenolic compounds, including rosmarinic acid and other hydroxycinnamic derivatives, which are considered among the principal bioactive constituents of sage.

The extract was manufactured in Italy, and the authors are not aware of any proprietary or patented extraction procedure associated with this specific commercial extract. The XenoScreen XL YES/YAS (Art. No. N04-233-Y) was obtained from Xenometrix AG (Gewerbestrasse 25, CH-4123 Allschwil, Switzerland); rosmarinic acid, 3-(4,5-dimethylthiazol-2-yl)-2,5-diphenyltetrazoliumbromide (MTT, Cat. Number M5655), 0.4% trypan blue dye (Cat. Number T8154), and 0.5% crystal violet (C0775) were purchased from Sigma-Adrich (Milan, Italy); and MCF-7 cells (human breast cancer cell line) were purchased from the American Type Culture Collection (ATTC, Manassas, VA, USA).

### 2.2. In Vitro Enzymatic Activity

Preparation of solutions: The estradiol standard was prepared according to the kit protocol. For analytical item preparation, a weighted amount of *Salvia officinalis* L. extract was dissolved in ultrapure water; the solution was allowed to stir for 15 min on a magnetic stirrer and then diluted 1:10 with a 1% DMSO solution in sterile water to the following final concentrations: 0.007 mg/mL, 0.014 mg/mL and 0.07 mg/mL.

Preparation and dispensing of yeast: The disc containing the yeast was placed with 5 mL of growth medium inside a sterile T25 flask. The flask was sealed with a vented filter cap and transferred to a shaking incubator saturated with double-distilled water set at 50 rpm at 31 °C for 3 days. After three days, 200 μL of the culture was sampled and analyzed to ensure that the yeast count was sufficient to perform the experiment. Optical density (OD_690_) was 0.5, which meets the OD ≥ 0.2 criterion established by the protocol. The suspension was then diluted 1:1 with the test medium, and 40 μL of this suspension was dispensed into each well.

Well dispensing: The wells were dispensed as shown in the following scheme (each analyte was seeded in duplicate).

The plate was incubated for at least 18 h in an orbital shaker set to 31 °C at 50 rpm. After 18 h, the plate was read at 570 and 690 nm on the plate reader (pre-lysis reading).

Preparation of lacZ reaction mixture and plate reading: The lacZ reaction mixture was prepared according to the manufacturer’s instructions. Briefly, 50 μL of reaction mixture was added to each well. Within 60 s of adding the buffer, an initial absorbance reading was recorded at 570 and 670 nm (60 s reading). The plate was then subsequently incubated at 31 °C in a shaking incubator set at 50 rpm for one hour, after which a second absorbance reading was performed at 570 and 670 nm (post-lysis reading). Estrogenic activity was then calculated according to the manufacturer’s protocol. For all spectrophotometric assays involving botanical preparations, a residual risk of matrix interference cannot be completely excluded; however, the controls employed indicate that the reported effects are unlikely to be attributable to optical artifacts.

### 2.3. Cell Culture

MCF-7 cells (human breast cancer cell line) from the American Type Culture Collection (Manassas, VA, USA) were cultured in MEM medium (Euroclone, Milan, Italy) containing 100 U/mL of penicillin and 100 μg/mL streptomycin (Euroclone, Milan, Italy), 2 mM L–glutamine (Euroclone, Milan, Italy) and 10% *v*/*v* fetal bovine serum (FBS; Invitrogen, Thermo Fisher Scientific, Milan, Italy). The cell culture was maintained in a humidified incubator at 37 °C in an atmosphere of 5% CO_2_.

### 2.4. Cell Viability Assay

MCF-7 cells (1 × 10^5^ cells/well) were seeded in 96-well plates and exposed to increasing concentrations of *Salvia officinalis* L. extract (from 0.0014 to 0.7 mg/mL) or rosmarinic acid (RA; Sigma-Adrich) (from 23.2 nM to 11.6 μM) for 4–72 h. Vehicle-treated cells served as control cells, which consisted of water for *Salvia Officinalis* L. extract and DMSO for RA. Cell viability was assessed using the 3-(4,5-dimethylthiazol-2-yl)-2,5-diphenyltetrazoliumbromide (MTT; Sigma-Aldrich, Milan, Italy) test. Briefly, after different times of incubation (4 to 72 h), the cell medium was replaced with fresh medium containing the MTT solution (1:10 *v*/*v*; Sigma-Aldrich) and the cells were incubated for 2 h at 37 °C. An MTT solubilization solution (0.1 N HCl containing 10% *v*/*v* Triton X-100 in anhydrous isopropanol) was used to dissolve the formazan crystals formed during the incubation by the activity of mitochondrial dehydrogenases of viable cells, and a microplate reader (DTX880 Multimode Detector, Beckman Coulter, Brea, CA, USA) was used to measure the absorbance of the cell culture medium at 570 nm. Experiments were performed in three independent biological replicates, each conducted in technical triplicate. Results were expressed as percentage of OD relative to vehicle-treated control cells. Half-maximal inhibitory concentration (IC_50_) values were calculated through the best fitting of dose–response curves using Graph Pad Prism 9 software (Graph Pad Software, San Diego, CA, USA).

### 2.5. Trypan Blue Exclusion Assay

MCF-7 cells were seeded (20 × 10^4^ cells per well) in 24-well plates for 24 h. Following that, the culture media was substituted with new medium that included different concentrations of *Salvia officinalis* L. extract (0.0014–0.7 mg/mL) that were incubated for 4–72 h at 37 °C. After these times, the cells were collected and diluted with 0.4% trypan blue dye (Sigma-Aldrich) at a 1:1 ratio and incubated for a few minutes before being counted using a Burker chamber. The viability was reported as the cell number. The study was conducted in biological triplicate, and each biological replicate was performed in technical triplicate. Data analysis was executed using Graph Pad Prism 9 software.

### 2.6. BrdU Cell Proliferation Assay

DNA synthesis, used as an indicator of cell proliferation, was assessed using a colorimetric bromodeoxyuridine (BrdU) incorporation Calbiochem QIA58 kit (Merck Millipore/Sigma-Aldrich, Cat. Number QIA58-200TEST). MCF-7 cells were seeded (10 × 104 cells per well) in 96-well plates for 24 h. The following day, cells were treated with different concentrations of *Salvia officinalis* L. extract (0.0014–0.7 mg/mL) for 4–72 h. At the end of treatment, cells were incubated with BrdU labeling solution (1:12,000) for 2 h at 37 °C. Subsequently, cells were fixed and permeabilized, and the DNA was denatured using fixative/denaturation solution. Cells were then incubated with an anti-BrdU antibody (1:100) for 1 h at room temperature (RT); unbound antibody was removed by washing, and horseradish peroxidase (HRP)-conjugated antibody was added. Following washes to remove excess conjugate, tetramethylbenzidine (TMB) substrate was added for 15 min at RT, the reaction was terminated using the stop solution containing 1 M H_2_SO_4_, and absorbance was measured spectrophotometrically at 450 nm. All experiments were performed in triplicate.

### 2.7. Colony-Forming Assay

The clonogenic potential of MCF-7 cells was evaluated using a colony-forming assay. Cells were seeded in 12-well plates at a density of 0.2 × 10^2^ cells/well in complete growth medium and allowed to attach for 18 h. The medium was then replaced with fresh complete growth medium containing the sage extract at concentrations ranging from 0.0014 to 0.7 mg/mL or vehicle alone (control). Cells were subsequently cultured for 9 days under standard incubation conditions without disturbance to allow colony formation. At the end of the incubation period, colonies were gently washed with phosphate-buffered saline (PBS; Na_2_HPO_4_/NaH_2_PO_4_, Euroclone, Milan, Italy), fixed with 10% neutral buffered formalin (Leica biosystems, Richmond, IL, USA) for 5 min, and stained with 0.5% crystal violet in methanol (C0775, Sigma-Aldrich) for 5 min at room temperature. Excess stain was removed by washing, and plates were allowed to air-dry. Colony formation was quantified using ImageJ software (version 1.54t; National Institutes of Health, Bethesda, MD, USA). Only colonies containing at least 50 cells were included in the analysis, using the corresponding pixel area as the acquisition threshold for image quantification.

### 2.8. Human Estrogen Receptor α (ERα) Reporter Gene Assay

Estrogenic activity was evaluated using the Human Estrogen Receptor Alpha (ERα) Reporter Assay System (Product number IB00401-32, Indigo Biosciences, State College, PA, USA), according to the manufacturer’s instructions. The assay is based on proprietary cryopreserved mammalian reporter cells constitutively expressing the full-length human estrogen receptor α together with a firefly luciferase reporter gene under the control of an estrogen-responsive promoter. Ligand-dependent activation of ERα induces transcription of the luciferase reporter gene, allowing quantitative assessment of receptor activation by luminescence. Briefly, reporter cells were thawed and dispensed at 100 μL/well into the assay plate and immediately treated with 100 μL of appropriate treatments; sage extract was tested at concentrations of 0.007, 0.014, 0.07 and 0.14 mg/mL in Compound Screening Medium. Vehicle-treated cells served as the negative control, whereas 17β-estradiol, supplied with the kit, was used as the positive control. For agonist-mode experiments, reporter cells were treated with scalar concentrations of the agonist 17β-estradiol to generate the reference dose–response curve and verify assay performance. For antagonist-mode experiments, a dose–response curve was generated by co-treating reporter cells with 17β-estradiol at a fixed dose of 3.2 nM (corresponding to the EC_75_ value) together with increasing concentrations of the selective antagonist, 4-hydroxytamoxifen.

Cells were incubated with treatments for approximately 24 h at 37 °C in a humidified atmosphere containing 5% CO_2_.

Following incubation, the treatment medium was removed and Luciferase Detection Reagent (100 μL/well) was added according to the manufacturer’s protocol. After the recommended stabilization period, luminescence was measured using a microplate luminometer TECAN and expressed as relative light units (RLUs). ERα activation was determined by normalizing luminescence values to vehicle-treated controls and expressed as fold activation induced by 17β-estradiol, used for the maximal receptor activation. Each experimental condition was analyzed in at least three independent experiments, each performed with technical replicates.

## 3. Results

### 3.1. In Vitro Enzymatic Activity

The concentrations of Salvira™ and rosmarinic acid were selected based on three main considerations: (i) ranges commonly employed in the literature for investigating estrogen receptor-mediated effects of botanical extracts and phytochemicals; (ii) preliminary cytotoxicity studies, which identified concentrations compatible with acceptable cell viability; and (iii) the need to cover a sufficiently wide concentration range to identify potential dose–response relationships. This approach enabled the evaluation of estrogenic activity under conditions that were both biologically relevant and experimentally informative, minimizing the risk that negative findings could be attributable to inappropriate concentration selection.

The concentrations of Salvira™ (sage standardized extract) were selected to reflect physiologically relevant ranges commonly employed in the literature. Specifically, doses of 280 mg and 560 mg of botanical extract were considered and divided by the theoretic physiologic fluid volume (40 L), obtaining concentrations of 0.007 mg/mL and 0.014 mg/mL. A ten-fold higher concentration was also tested to determine LoQ and LoD (0.07 mg/mL).

The data obtained were processed following the formulas indicated in the protocol for calculating estrogenic activity. The following parameters were calculated:

$$\begin{array}{cc} Growth\;Factor\;G & G=\frac{A_{690},S}{A_{690},N}\\ Beta-galactosidase\;activity(relativeunits)US: & Us=\frac{A_{570},S}{A_{690},S}\\ Induction\;ratio\;Ir & Ir=\frac{1}{G}*\frac{A_{570},S}{A_{570},N} \end{array}$$

where

- A_690_, S is the absorbance of sample S at 690 nm before lysis.
- A_690_, N is the absorbance of the solvent control at 690 nm before lysis.
- A_570_, S is the net absorbance of sample S calculated as absorbance at 570–690 nm after lysis.
- A_570_, N is the net absorbance of the solvent control calculated as absorbance at 570–690 nm after lysis.
- Ir (induction ratio) is the value indicating the induction effect of the substance on the estrogen receptor. A sample Ir value ≥ Ir10 indicates an agonistic effect (where Ir10 is defined as the Ir that is 10% of Ir estradiol-Ir solvent).

Based on the Ir values obtained and reported in Table 1, *Salvia officinalis* L. extract did not exhibit estrogenic activity at any tested concentration, as their respective sample Ir values are lower than the Ir10 value. In contrast, the pure reference agonist standard, as can be observed, has an Ir value between 15 and 20 times higher than that of the tested samples and more than 10 times greater than Ir10.

### 3.2. Cell Viability

**Figure 1:**
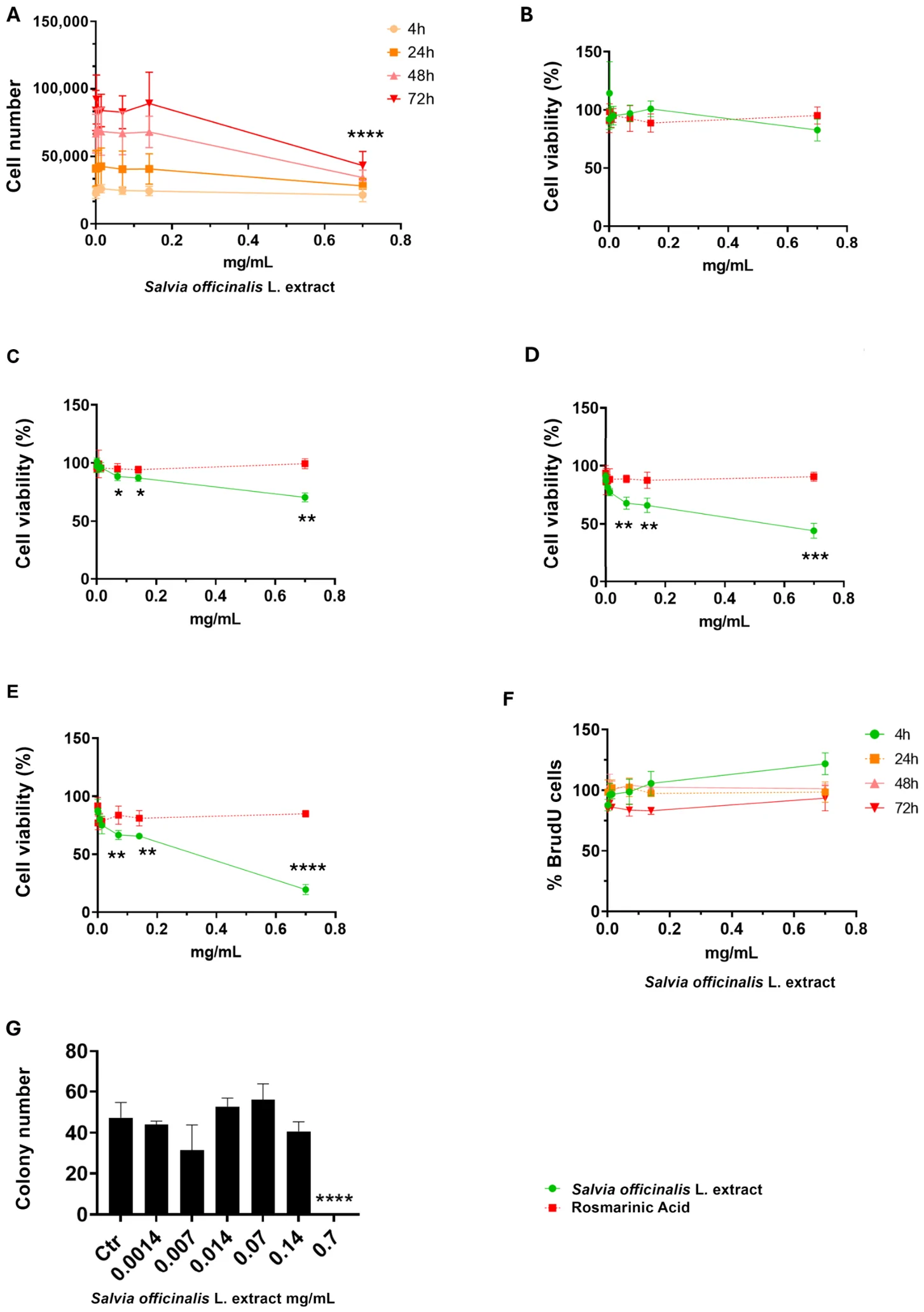
Effects of *Salvia officinalis* L. extract on MCF-7 cell viability, proliferation, and clonogenic capacity. (**A**) Number of viable cells determined by the trypan blue exclusion assay after 4, 24, 48, and 72 h of treatment with increasing concentrations of *S. officinalis* L. extract. One-way ANOVA: Ctr vs. all treatments; **** *p* < 0.0001. (**B**–**E**) MCF-7 cell viability, expressed as a percentage of the untreated control, assessed by the MTT assay after 4 h (**B**), 24 h (**C**), 48 h (**D**), and 72 h (**E**) of exposure to *S. officinalis* L. extract or rosmarinic acid. t-test: Ctr vs. all treatments; * *p* < 0.05; ** *p* < 0.01; *** *p* < 0.001; **** *p* < 0.0001. (**F**) Cell proliferation evaluated by BrdU incorporation after 4, 24, 48, and 72 h of treatment with increasing concentrations of the extract; results are expressed as a percentage of BrdU positive cells. (**G**) Clonogenic capacity of MCF-7 cells following treatment with increasing concentrations of *S. officinalis* L. extract, expressed as the number of colonies formed. Data are presented as mean ± SD. One-way ANOVA: Ctr vs. all treatments: **** *p* < 0.0001 versus untreated control.

Cell viability was evaluated using both the trypan blue exclusion and MTT assays. The number of MCF-7 cells progressively increased over the 4–72 h incubation period; however, exposure to increasing concentrations of *Salvia officinalis* L. extract resulted in a dose- and time-dependent reduction in cell number and metabolic viability (Figure 1A–E). This effect was most pronounced at the highest concentration tested (0.7 mg/mL), at which a marked decrease in both viable cell number (Figure 1A) and MTT signal was observed, suggesting an adverse effect on cell survival and/or growth viability (Figure 1B–E). In contrast, rosmarinic acid administered alone did not significantly affect MCF-7 cell viability, with values remaining comparable to those of untreated controls (Figure 1B–E). Overall, these findings indicate that the reduction in cell viability induced by the complete extract cannot be attributed to rosmarinic acid alone and may instead result from the additive or synergistic effects of multiple bioactive constituents present in the phytocomplex.

### 3.3. BrdU Cell Proliferation Assay

Overall, the extract did not exert a substantial inhibitory effect on cell proliferation at the concentrations tested. The BrdU incorporation values remained close to 100% of the control, indicating only a limited effect on DNA synthesis. Consistent with these findings, the BrdU assay demonstrated that the *Salvia officinalis* L. extract did not significantly affect MCF-7 cell proliferation (Figure 1F). Therefore, despite the reduction in cell viability observed in the previous assays, DNA synthesis remained largely preserved, suggesting that the effects of the extract are more likely associated with cytotoxicity and/or metabolic alterations rather than with a direct inhibition of cell proliferation.

### 3.4. Colony-Forming Assay

Treatment with *Salvia officinalis* L. extract did not affect colony formation at concentrations up to 0.14 mg/mL, indicating that the long-term clonogenic capacity of the cells was preserved within this concentration range. In contrast, exposure to 0.7 mg/mL, the highest concentration tested, completely suppressed colony formation, demonstrating a profound reduction in reproductive cell survival (Figure 1G). These findings suggest a concentration-dependent cytotoxic effect, with the highest tested concentration markedly impairing the cells ability to proliferate and generate colonies.

### 3.5. ERα Agonist-Mode Dose–Response Analysis Assay

**Figure 2:**
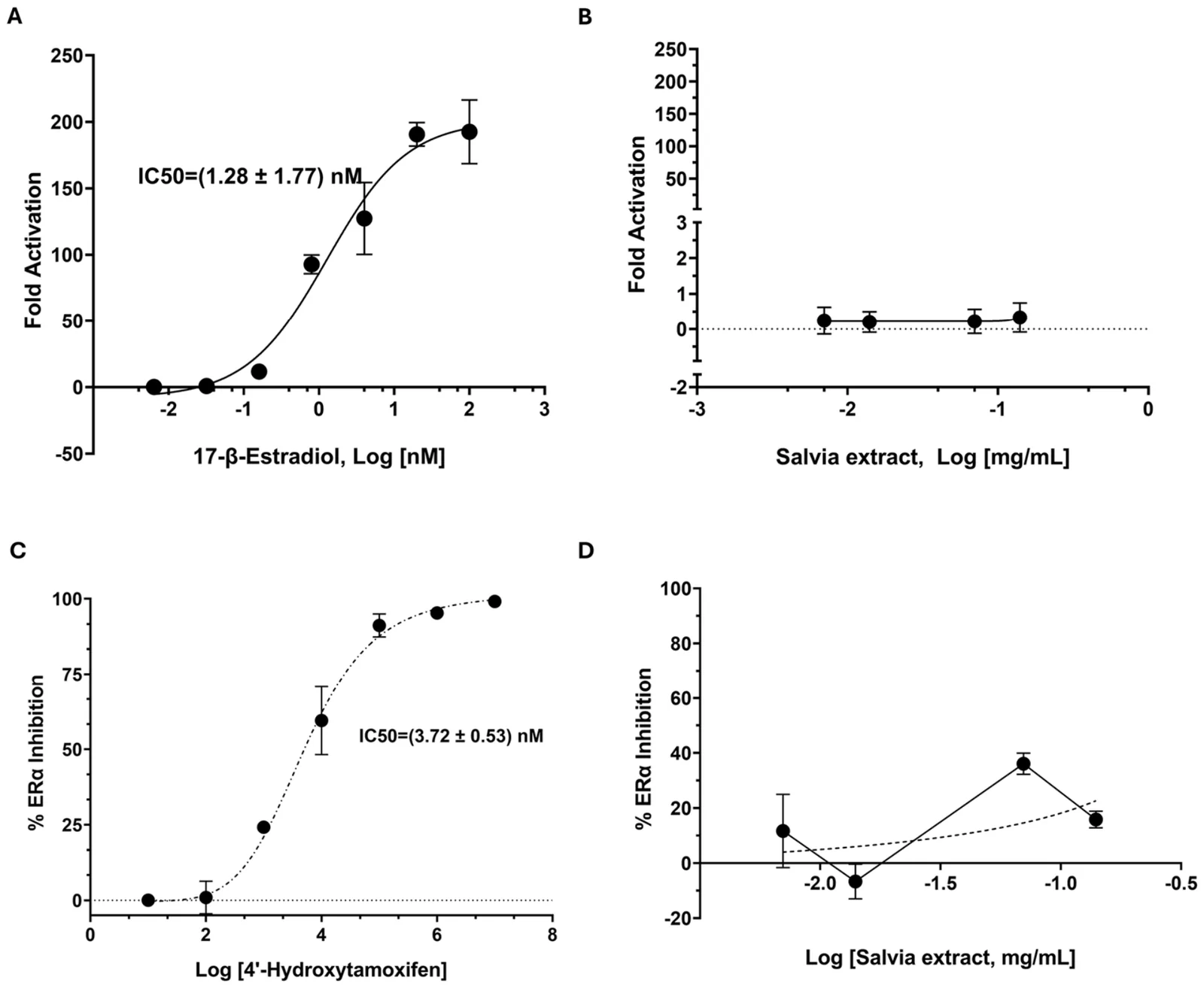
Evaluation of the estrogenic and anti-estrogenic activity of *Salvia officinalis* L. extract using an ERα reporter assay. (**A**) Concentration–response curve for the ERα agonist reference compound 17β-estradiol, expressed as fold activation, with an EC_50_ value of 1.28 ± 1.77 nM. (**B**) ERα activation following exposure to increasing concentrations of *S. officinalis* L. extract, showing no relevant agonist activity over the tested concentration range. (**C**) Concentration–response curve for the ERα antagonist reference compound 4′-hydroxytamoxifen, expressed as percentage inhibition of ERα activity, with an IC_50_ value of 3.72 ± 0.53 nM. (**D**) Percentage inhibition of ERα activity following treatment with increasing concentrations of *S. officinalis* L. extract. Data are presented as mean ± SD.

This experiment was conducted to assess the potential estrogenic activity of the sage extract using an ERα agonist-mode dose–response assay. The assay evaluates the ability of the test substance to activate estrogen receptor alpha (ERα) in a concentration-dependent manner and thereby determines whether it exhibits agonistic activity toward the receptor. A range of extract concentrations was tested, and the resulting responses were compared with those elicited by the reference estrogenic agonist 17-β-Estradiol, used as a positive control (Figure 2A). This experimental approach enables the characterization of both the potency and efficacy of the extract and provides important information on its potential endocrine activity and safety profile. Different concentrations of the *Salvia officinalis* L. extract were also tested to determine whether they induce activation of ERα in a concentration-dependent manner. The responses were compared with those obtained using a reference estrogen receptor agonist, allowing us to evaluate the estrogenic potency of the extract and its suitability as a safe botanical ingredient.

The results demonstrated that *Salvia officinalis* L. extract showed no estrogenic activity across the tested concentration range. No ERα activation was detected, indicating that the extract does not act as an ERα agonist (Figure 2B). These findings suggest the absence of estrogen receptor-mediated activity and support the safety profile of the extract.

### 3.6. ERα Antagonist-Mode Dose–Response Analysis Assay

The ERα antagonist-mode assay showed that *Salvia officinalis* L. extract did not exert ERα at any of the concentrations tested (Figure 2D). ERα antagonist reference compound 4′-hydroxytamoxifen was used as a positive control (Figure 2C). The ERα antagonist-mode assay showed that *Salvia officinalis* L. extract did not exert antagonistic effects at any of the concentrations tested. No inhibition of agonist-induced ERα activation was observed, confirming that the extract does not act as an ERα antagonist (Figure 2D). When considered alongside the agonist-mode data, these results indicate a lack of estrogen receptor-mediated activity and corroborate the endocrine safety of the extract.

## 4. Discussion

The present study investigated whether a standardized *Salvia officinalis* L. extract exerts estrogen receptor-mediated activity, with particular emphasis on ERα signaling and estrogen-responsive breast cancer cells. This question is relevant because sage-containing food supplements are increasingly used for the management of menopausal vasomotor symptoms, whereas the mechanisms underlying their effects remain incompletely defined. Although clinical studies have reported reductions in hot-flush frequency and severity following supplementation with *S. officinalis* preparations [14,22,26], it is still unclear whether these benefits involve estrogen receptor activation or alternative, non-hormonal pathways.

The results obtained in the present investigation consistently indicate the absence of detectable ERα-mediated estrogenic activity of the tested extract under the experimental conditions employed. Specifically, neither the XenoScreen XL YES/YAS assay nor the mammalian ERα reporter gene assay provided evidence of estrogen receptor activation or inhibition, suggesting that the extract does not function as a direct ERα agonist or antagonist.

These observations are particularly relevant considering previous reports suggesting possible estrogenic properties of sage extracts. Certain in vitro studies have attributed estrogen receptor interactions to specific flavonoid constituents, particularly luteolin derivatives, which were shown to exhibit weak binding affinity toward estrogen receptors [23,24]. However, other investigations have failed to reproduce such findings, highlighting substantial variability among sage preparations and emphasizing the influence of extraction procedures, geographical origin, phytochemical composition, and standardization parameters [25,26]. The discrepancy between studies may therefore reflect differences in extract composition rather than intrinsic estrogenic properties of the plant itself.

The lack of estrogen receptor activation was further supported by the results obtained in MCF-7 breast cancer cells, a widely accepted model for studying estrogen-responsive signaling. Estrogenic compounds typically stimulate cell proliferation and DNA synthesis in MCF-7 cells through ER-dependent mechanisms. In contrast, the tested *Salvia officinalis* extract did not promote cell growth under any experimental condition. Rather, both the trypan blue and MTT assays demonstrated a concentration-dependent reduction in cell viability, while the BrdU assay showed no significant increase in DNA synthesis. Collectively, these findings provide further evidence against the presence of proliferative estrogen-like effects.

Interestingly, the discrepancy between the reduction in cell viability and the relatively preserved BrdU incorporation suggests that the extract may affect cellular metabolism or induce cytotoxic responses without directly altering cell-cycle progression at lower concentrations. Similar effects have been described for polyphenol-rich botanical extracts, whose impact on mitochondrial function may precede detectable changes in DNA synthesis. The maintenance of BrdU incorporation at physiologically relevant concentrations indicates that the extract neither stimulates nor substantially inhibits cell proliferation, supporting a neutral profile with respect to estrogen-responsive pathways.

The clonogenic assay provided additional information on long-term cellular survival. Colony formation was preserved up to 0.14 mg/mL, whereas complete inhibition was observed only at the highest concentration tested. This finding is consistent with the cytotoxic effects observed in short-term viability assays and is more likely attributable to non-specific cellular toxicity than to estrogen receptor modulation. Nevertheless, in the absence of pharmacokinetic data, caution is warranted when extrapolating in vitro concentrations to human exposure conditions.

Taken together, these observations align with the current understanding of botanical extracts as complex phytochemical matrices whose pharmacological effects emerge from the interaction of numerous constituents acting on multiple molecular targets. Since estrogen receptor activation appears unlikely, the therapeutic effects observed in clinical studies may instead involve alternative pathways. Several mechanisms have been proposed, including modulation of central thermoregulatory networks, inhibition of acetylcholinesterase activity, serotonergic and GABAergic neurotransmission, and the traditional antiperspirant properties attributed to sage constituents [20,21]. Such mechanisms are biologically plausible given the current understanding of vasomotor symptom pathophysiology, which increasingly recognizes the importance of hypothalamic neurotransmitter dysregulation beyond estrogen deficiency alone.

Overall, the integration of yeast-based screening, mammalian ERα reporter assays, and functional studies in estrogen-responsive MCF-7 cells provides a coherent body of evidence that the investigated *S. officinalis* L. extract does not exert direct agonistic or antagonistic activity toward ERα. Within the limits of the experimental design, these findings support a non-estrogenic mode of action and contribute to the endocrine safety characterization of this standardized botanical ingredient.

In contrast, several well-characterized phytoestrogenic botanicals, including soybean (*Sojae semen*), flaxseed (*Lini semen*), and sesame (*Sesami semen*), have demonstrated measurable estrogenic activity in experimental models. Soy is rich in isoflavones such as genistein and daidzein, whereas flaxseed and sesame contain lignans and their metabolites, all compounds capable of interacting with estrogen receptors and modulating estrogen-responsive pathways [27].

The phytochemical profile of *Salvia officinalis* differs substantially from these materials. Sage is characterized mainly by phenolic acids, particularly rosmarinic acid, diterpenes such as carnosic acid and carnosol, flavonoids, and volatile constituents, while lacking the high concentrations of isoflavones or lignans typically associated with direct estrogen receptor activation [19].

Therefore, the discrepancy between previous claims of estrogenic activity and the present findings may reflect differences in extract composition, plant origin, extraction procedures, standardization criteria, and experimental models. In contrast to classical phytoestrogens, the standardized sage extract investigated here did not directly modulate estrogen receptor signaling, suggesting that its reported benefits in women’s health may instead rely on alternative mechanisms, including antioxidant, anti-inflammatory, neuroprotective, and metabolic activities [19].

Taken together, a comparison with well-established phytoestrogenic botanicals such as soybean, flaxseed, and sesame highlights an important distinction: while these materials contain recognized estrogen receptor ligands, the extract investigated in the present study was obtained through a standardized manufacturing process, which may partially explain the differences observed compared with previous reports obtained using non-standardized or compositionally distinct sage preparations.

Nevertheless, some limitations should be acknowledged. The absence of estrogenic activity in the in vitro models employed does not completely exclude the possibility that metabolites generated following oral administration could exert biological effects in vivo. At the same time, the lack of direct estrogen receptor activation or inhibition observed across multiple experimental systems suggests that any physiological benefits associated with the extract are unlikely to be mediated by a classical estrogen receptor agonist mechanism. Further comprehensive phytochemical characterization of the extract, clinical studies, and pharmacokinetic investigations will be required to determine whether the non-estrogenic profile observed in vitro is maintained under physiological conditions in humans.

Therefore, caution should be exercised when classifying *Salvia officinalis* as a phytoestrogen solely on the basis of traditional use, phytochemical assumptions, or indirect biological observations. Rather, the present findings support the view that the beneficial effects of standardized sage extracts may predominantly involve non-estrogenic pathways and mechanisms independent of direct estrogen receptor modulation.

## 5. Conclusions

In conclusion, the present findings suggest that the standardized *Salvia officinalis* L. extract Salvira™ does not directly modulate ERα signaling under the experimental conditions investigated, supporting a non-estrogenic mode of action. These results contribute to the safety characterization of this ingredient and indicate that the beneficial effects reported for sage preparations in menopausal women are likely mediated through mechanisms other than direct estrogen receptor activation.

From an industrial perspective, the long history of dietary use of sage, together with the absence of detectable ERα-mediated activity observed in the present study, supports the development of standardized sage extracts as ingredients for functional foods and nutraceutical products targeting women’s health and menopausal well-being. Future well-designed human studies will be required to confirm whether the non-estrogenic profile observed in vitro is maintained under physiological conditions and to better define the role of standardized *Salvia officinalis* preparations in the management of menopausal symptoms.

## Figures and Tables

**Table 1:** Data obtained from the calculations of G, Us and Ir values, using the averages of the duplicate values.

Sample Dose	G	Us	Ir	Ir 10
0.007 mg/mL	1.835157	0.259986	0.45 ± 0.04	0.801817
0.014 mg/mL	1.768817	0.307994	0.53 ± 0.05	
0.07 mg/mL	1.934292	0.308706	0.54 ± 0.05	
DMSO 1%	1		1 ± 0.1	
estradiol 0.00068 mg/mL	1.006088		9.01 ± 0.9	

## Data Availability

The data that support the findings of this study are available from the corresponding author on reasonable request.
